# Population-based comparison of post-acute sequelae of COVID-19 and health-related quality of life across pandemic periods: Omicron era versus early pandemic

**DOI:** 10.1038/s41598-026-52945-2

**Published:** 2026-05-19

**Authors:** Raphael S. Peter, Alexandra Nieters, Lisamaria Sedelmaier, Hans-Georg Kräusslich, Stefan O. Brockmann, Siri Göpel, Uta Merle, Jürgen M. Steinacker, Dietrich Rothenbacher, Winfried V. Kern

**Affiliations:** 1 Institute of Epidemiology and Medical Biometry, Ulm University, 89081 Ulm, Germany; 2 Institute for Immunodeficiency, Medical Centre and Faculty of Medicine, Albert-Ludwigs-University, Freiburg, Germany; 3 Department of Infectious Diseases, Virology, Heidelberg University, Heidelberg, Germany; 4 Department of Health Protection, Infection Control and Epidemiology, Baden-Wuerttemberg Federal State Health Office, Ministry of Social Affairs, Health and Integration, Stuttgart, Germany; 5 Department of Internal Medicine I, University Hospital Tübingen, Tübingen, Germany; 6 Department of Internal Medicine IV, University Hospital Heidelberg, Heidelberg, Germany; 7 Institute of Rehabilitation Medicine Research at Ulm University, Ulm, Germany; 8 Division of Sports and Rehabilitation Medicine, Department of Medicine, Ulm University Hospital, Ulm, Germany; 9 Division of Infectious Diseases, Department of Medicine II, Medical Centre and Faculty of Medicine, Albert-Ludwigs-University, Freiburg, Germany; 10 Department of Health, Heidenheim District Administration Office, Heidenheim, Germany; 11 Department of Health, Zollernalbkreis District Administration Office, Balingen, Germany; 12 Department of Health, Alb-Donau-Kreis District Administration Office, Ulm, Germany; 13 Institute for Exercise and Occupational Medicine, Medical Centre and Faculty of Medicine, Albert-Ludwigs-University, Freiburg, Germany; 14 Department of Health, Emmendingen District Administration Office, Emmendingen, Germany; 15 Department of Sports Medicine, Heidelberg University Faculty of Medicine and Heidelberg University Hospital, Heidelberg, Germany; 16 Department of Health, Tübingen District Administration Office, Tübingen, Germany; 17 Department of Health, Breisgau-Hochschwarzwald District Administration Office, Freiburg, Germany; 18 German Center for Mental Health (DZPG), Partner Site Mannheim-Heidelberg-Ulm, Mannheim, Germany; 19 Clinical & Biological Psychology, Institute of Psychology and Education, Ulm University, Ulm, Germany; 20 Department of Sports Medicine, University Hospital Tübingen, Tübingen, Germany; 21 Ministry of Social Affairs, Health and Integration, Stuttgart, Germany; 22 Department of Psychiatry and Psychotherapy, Medical Faculty Mannheim, Central Institute of Mental Health (ZI), University of Heidelberg, Mannheim, Germany; 23 Department of Health, Rhein-Neckar-Kreis District Administration Office, Heidelberg, Germany; 24 Department of Health, Biberach District Administration Office, Biberach, Germany; 25 Department of Health, Reutlingen District Administration Office, Reutlingen, Germany

**Keywords:** Post-COVID-19 syndrome, Long COVID, SARS-CoV-2 variants, Omicron, Health-related quality of life, Diseases, Health care, Medical research, Microbiology

## Abstract

Post-COVID-19 syndrome (PCS) may vary across pandemic phases. We compared the prevalence of post-acute COVID-19 symptom clusters following SARS-CoV-2 infection in the early pandemic and during the Omicron era using a population-based approach. The EPILOC study enrolled individuals aged 18–65 years who tested PCR-positive for SARS-CoV-2 during the early pandemic, between October 2020 and April 2021, in defined geographic regions within Baden-Württemberg. EPILOC-Omicron used an identical design for individuals infected between June and July 2022. Participants completed a standardised questionnaire assessing sociodemographics, lifestyle, symptoms, and health-related quality of life (SF-12). PCS was defined as ≤ 80% recovery of general health or work capacity plus at least one new moderate-to-strong symptom, both compared to before index infection. Generalised linear models adjusted for age, sex, and education were used to estimate relative risks (RRs). We analysed data from 11,710 EPILOC (24% response) and 12,560 EPILOC-Omicron participants (17% response). Participants were similar in age and sex distribution. Vaccination before infection differed markedly (< 3 vs. > 92%). PCS prevalence was 29.6% in EPILOC and 14.5% in EPILOC-Omicron. Predictors of PCS were similar for both. Symptom clusters were consistently less frequent after infection during the Omicron era (e.g., fatigue RR = 0.54; neurocognitive impairment RR = 0.53; chest symptoms RR = 0.47; smell/taste disorder RR = 0.17). Health-related quality of life was similar in PCS cases of both cohorts (mean SF-12_Physical_ 40.3 vs. 41.0, mean SF-12_Mental_ 38.9 vs. 40.7). PCS was less common following SARS-CoV-2 infection in the Omicron era compared to the early pandemic period. However, affected individuals continue to experience substantial, comparable impairment in hrQoL across both pandemic periods.

**Supplementary Information:**

The online version contains supplementary material available at 10.1038/s41598-026-52945-2.

## Introduction

Post-COVID-19 syndrome (PCS), characterised by persistent symptoms following acute SARS-CoV-2 infection, has emerged as a major health concern during the pandemic. While the clinical burden of PCS is well recognised, questions remain about how the risk and presentation of PCS may vary with different viral variants. In particular, as the Omicron variant became globally dominant, it has been suggested that the frequency and severity of PCS differs compared to infections with earlier variants such as the wild-type virus.

Omicron, first identified in late 2021, has been associated with a generally milder acute disease course compared to earlier variants, particularly among vaccinated populations^1^. Nevertheless, the long-term consequences of Omicron infection remain poorly understood. Given the widespread transmission of Omicron and its subvariants, understanding the variant-specific risk of developing PCS is of significant public health importance.

Previous research suggests that variant-specific differences in viral characteristics, host immune responses, and background immunity, due to vaccination or prior infection, may affect the risk of PCS^2–4^. However, direct comparisons of PCS prevalence following infection across pandemic phases with different variants, based on standardised population-based approaches, are still limited. Most available data derive from hospitalised cohorts or unrepresentative samples^5–7^, complicating interpretation and generalisability. Although comparisons based on electronic health records exist^3^, these data are, however, usually insufficient to capture the subjective symptom burden and impairments in health-related quality of life (hrQoL).

In this context, we conducted a population-based study comparing PCS prevalence and symptom patterns following infection during the early pandemic and Omicron era. Using two large cohorts within the same defined geographic regions from the federal state of Baden-Württemberg, Germany – EPILOC and EPILOC-Omicron – we applied the same study design, standardised questionnaires, and consistent case definitions for PCS. Our analysis aimed to test whether the risk of PCS differed between infections occurring in the early pandemic and during the Omicron era, and to characterise symptom clusters and quality of life impairments in affected individuals.

## Methods

EPILOC (Epidemiology of Long Covid) is a population-based longitudinal observational study conducted in the Federal State of Baden-Württemberg (southwestern Germany). The baseline assessment was conducted in 2021. Subjects between 18 and 65 years old who had a PCR-confirmed SARS-CoV-2 infection between 1st October 2020 and 1st April 2021 were enrolled six to 12 months after acute infection. The study population initially received a letter containing participation information, an informed consent form, and a standardised questionnaire directly from the local public health offices. Details of the EPILOC baseline assessment have been reported previously^8^. EPILOC-Omicron invited individuals of the same source population who were infected between 15. June and 15. July 2022 and used identical inclusion criteria. In Germany, at that time, local public health authorities had still to be notified of SARS-CoV-2 infection according to the German Infection Protection Act. However, due to limited resources at that time, not all infections could be PCR-confirmed.

This study was conducted in accordance with the Declaration of Helsinki. Ethical approval was obtained from the respective ethical review boards of the study centres at Albert-Ludwigs-University Freiburg (Approval No: 21/1484) and Ulm University (Approval No: 337/21).

### Data sources and measurements

EPILOC and EPILOC-Omicron questionnaires included identical questions about additional SARS-CoV-2 infections, vaccinations, general state of health, working capacity, and health-related quality of life (hrQoL) assessed using the Short-Form-Health Survey (SF-12). In addition, the questionnaires contained a list of 30 symptoms and asked for information about the occurrence of these symptoms before and during the index infection and at the time of questionnaire completion. The impairment of persistent or newly occurring symptoms at baseline or follow-up was assessed on a Likert-type scale (none, light, moderate, strong). Treatment of the acute infection was defined as any physician contact related to the acute infection.

### Statistical methods

PCS cases were defined, identical for both cohorts, as general health or working capacity recovered to a level no more than 80% (compared to pre-COVID-19 index infection), and any new symptom (excluding vomiting, nausea, stomach ache, diarrhoea, chills, fever) of grade moderate to strong regarding impairment in daily life and not already present before the acute index infection. The EPILOC PCS case definition was developed by the EPILOC consortium and has been applied in previous EPILOC analyses^9^. Compared to purely symptom-based definitions, it incorporates functional impairment and symptom severity, which are key elements of clinically relevant PCS. In addition, we applied the COVIDOM PCS case definition, which is based on a symptom score with symptom-specific weightings^10^. Symptom clusters were previously identified using data from the EPILOC baseline assessment^8^.

For prevalence comparisons between cohorts, we used generalised linear models adjusted for age, sex and education (university entrance qualification yes/no, i.e., completion of upper secondary education enabling university access) to estimate adjusted relative risks. Adjusted differences in symptom prevalence (Omicron era vs. early pandemic) during acute COVID-19 and for post-COVID-19 were modelled using binomial linear models adjusted for age, sex and education. To investigate potential effect modification, predictors of case status (including age, sex, education, smoking status, obesity, treatment of the acute infection, and preexisting diseases) for each cohort were modelled using generalised linear models, with mutual adjustment of covariates.

Differences in hrQoL of the mental and physical subdomains between PCS cases and PCS negatives adjusted for age, sex and education, were calculated using linear models for both cohorts. All reported confidence intervals are based on robust standard errors.

We did not perform any imputation for missing values (maximum number of missing were observed for hair loss as a symptom with 6%). Statistical procedures were performed with the SAS statistical software package (release 9.4, SAS Institute Inc.) or R version 4.5.0.

## Results

**Fig. 1:**
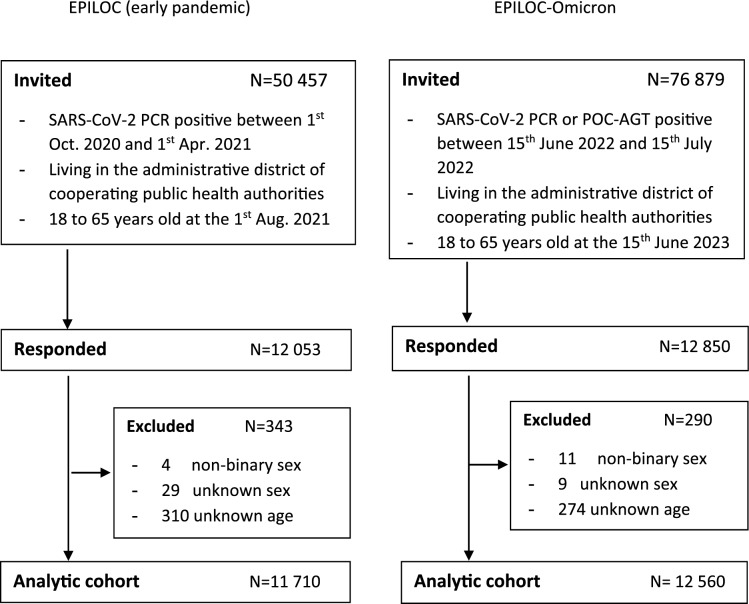
Flow chart.

Data of 11 710 participants from the EPILOC cohort (early pandemic) and 12 560 from the EPILOC-Omicron cohort could be used for analysis (Fig. 1). We previously estimated, based on national sequencing data, that 85% of the EPILOC cohort had been infected with the wild-type and about 15% might have been infected with the Alpha variant (B.1.1.7)^8^. For EPILOC-Omicron we estimated a contribution of 80% BA.5, 13% BA.2, and 7% BA.4 lineages during the index-infection period^11^.

The mean age of both cohorts was similar (45.9 vs. 44.1 years), while the proportion of women was slightly higher in EPILOC-Omicron (62.3%) compared to EPILOC (58.8%, Table 1). Participants in EPILOC-Omicron were more likely to have acquired a university entrance qualification (64.6 vs. 51.9%), were more likely to have never smoked (68.9 vs. 65.1%), and were less likely to be obese (15.8 vs. 18.7%). Fewer EPILOC-Omicron participants received medical treatment for the acute infection (17.8 vs. 22.5%). Of the EPILOC-Omicron participants, 76.8% reported no other known SARS-CoV-2 infection besides the index infection. More than 92% of the EPILOC-Omicron participants had received at least one vaccine dose, even when it was assumed that all participants who did not provide information on vaccination were unvaccinated. The mean time from the index infection was longer for EPILOC-Omicron than for EPILOC (15.1 vs 8.5 months).

**Table 1:** Characteristics of the study population.

		EPILOC (early pandemic)			EPILOC-Omicron	
		N		Number (%) or mean (sd)	N	Number (%) or mean (sd)
Age (years), mean (sd)		11 710		44.1 (13.7)	12 560	45.9 (13.2)
Age class (years), N (%)						
< 30		11 710		2474 (21.1)	12 560	2004 (16.0)
30–< 40				2158 (18.4)		2380 (19.0)
40–< 50				2075 (17.7)		2395 (19.1)
50–< 60				3443 (29.4)		3732 (29.7)
≥ 60				1560 (13.3)		2049 (16.3)
Sex, N (%)						
Male		11 710		4829 (41.2)	12 560	4730 (37.7)
Female				6881 (58.8)		7830 (62.3)
University entrance qualification						
Yes		11 678		6065 (51.9)	12 539	8100 (64.6)
No				5613 (48.1)		4439 (35.4)
Nationality						
German		11 688		11 004 (94.2)	12 549	12 174 (97.0)
Other^1^				684 (5.9)		375 (3.0)
Pre-pandemic employment						
Full time		11 628		6608 (56.8)	12 517	7055 (56.4)
Part time				3220 (27.7)		3659 (29.2)
Studying/vocational education				1143 (9.8)		1203 (9.6)
None				657 (5.7)		600 (4.8)
Smoking status, N (%)						
Current		11 678		1192 (10.2)	12 466	1187 (9.5)
Former				2882 (24.7)		2693 (21.6)
Never				7604 (65.1)		8586 (68.9)
BMI (kg/m^2^), mean (sd)		11 619		26.1 (5.3)	12 496	25.5 (4.9)
Obese (≥ 30 kg/m^2^), N (%)		11 619		2171 (18.7)	12 496	1978 (15.8)
Pre-existing conditions, N (%)						
Musculoskeletal disorders (including rheumatism)		11 448		3310 (28.9)	12 381	3329 (26.9)
Cardiovascular disorders (including hypertension)		11 477		1992 (17.4)	12 423	1882 (15.2)
Respiratory diseases		11 467		1385 (12.1)	12 402	1462 (11.8)
Mental disorders		11 479		1470 (12.8)	12 436	1663 (13.4)
Neurological or sensory disorders		11 480		1855 (16.2)	12 438	2170 (17.5)
Dermatological diseases		11 547		1257 (10.9)	12 410	1350 (10.9)
Cancer		11 323		386 (3.4)	12 357	480 (3.9)
Metabolic disorders		11 554		2014 (17.4)	12 438	2134 (17.2)
Times since positive test (months), mean (sd)		11 521		8.5 (1.6)	12 560	15.1 (0.7)
Treatment of acute SARS-CoV-2 infection						
No medical care		11 602		8988 (77.5)	12 382	10 184 (82.3)
Outpatient care				2202 (19.0)		2130 (17.2)
Inpatient care (without ICU)				315 (2.7)		57 (0.5)
Intensive care				97 (0.8)		11 (0.1)
Number of additional SARS-CoV-2 infections						
0		11 710		11 710 (100)	12 514	9610 (76.8)
1				0 (0.0)		2614 (20.9)
2				0 (0.0)		276 (2.2)
3				0 (0.0)		14 (0.1)
Number of vaccine dose against SARS-CoV-2 received before positive test, N (%)						
0		11 670		9387 (97.6)	11 694	17 (0.2)
1				^2^ 229 (2.4)		110 (0.9)
2				0 (0.0)		1078 (9.2)
3				0 (0.0)		10 102 (86.4)
4				0 (0.0)		387 (3.3)

^1^only includes participants without German citizenship.

^2^of which 159 were in the month of the positive test.

**Fig. 2:**
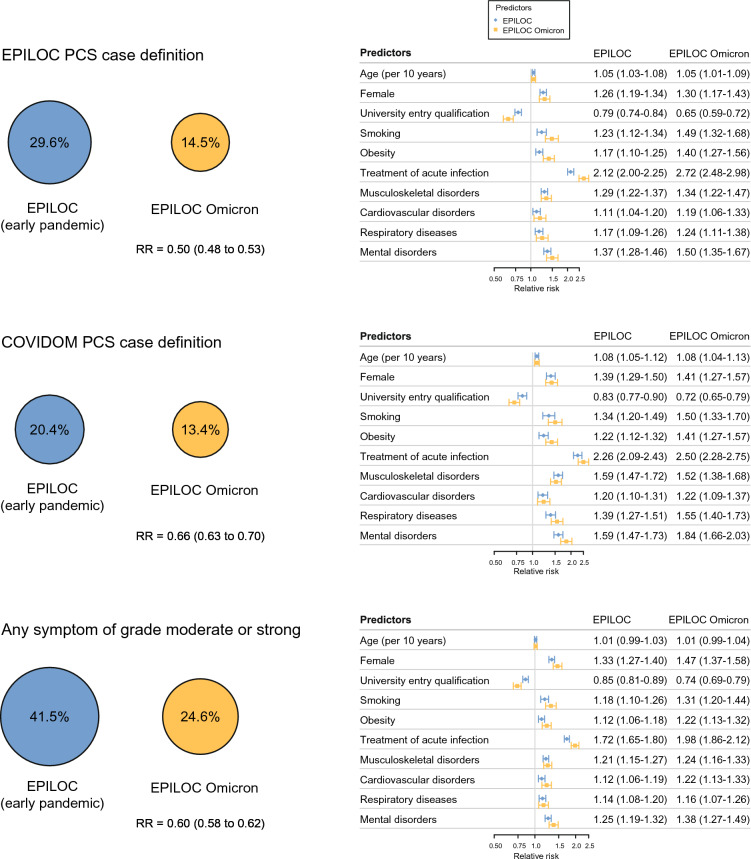
Prevalence of PCS according to the EPILOC and COVIDOM case definition in the EPILOC (early pandemic) and EPILOC-Omicron cohorts (left panel). The risk ratio (RR) for PCS after infection during Omicron vs. early pandemic is adjusted for age, sex and education. Mutually adjusted predictors of PCS case status (relative risk with 95% confidence interval) are shown in the right panel.

The EPILOC PCS case definition was fulfilled by 29.6% of EPILOC and 14.5% of EPILOC-Omicron (Fig. 2, left panel). When applying the COVIDOM case definition, the PCS prevalence was lower for EPILOC participants, but still higher compared to EPILOC-Omicron (20.4 vs. 13.4%). When PCS was defined as any (new) symptom of grade moderate to strong the prevalences were 41.5 vs. 24.6%, respectively. Associations of potential PCS predictors with case status were consistent across both cohorts and all case definitions (Fig. 2, right panel) and included treatment of the acute infection, female sex, lower education, current smoking, obesity, and pre-existing diseases.

With regard to specific symptoms and complaints, some of them were more often reported during the acute infection in the Omicron era (e.g., sore throat, cough, fewer, hoarseness, chills, and melalgia), whereas others were more common with the acute infection during the early pandemic (e.g., altered smell, altered taste, chest pain, chronic fatigue, diarrhea, anxiety, depressive mood, and memory disturbance, Supplemental Fig. 1). However, concerning post-COVID-19 symptoms, none of the symptoms were more common in the Omicron era compared to the early pandemic. The most pronounced risk difference between EPILOC compared to EPILOC Omicron was observed for altered smell (+ 16.2% for early pandemic) followed by altered taste (+ 12.5%), shortness of breath (+ 12.3%), rapid physical exhaustion (+ 12.1%), concentration difficulties (+ 10.0%), chronic fatigue (+ 8.5%), and memory disturbance (+ 8.3%). Prevalences of specific post-COVID-19 symptoms with grade of impairment can also be found in the supplement (Supplemental Fig. 2).

**Fig. 3:**
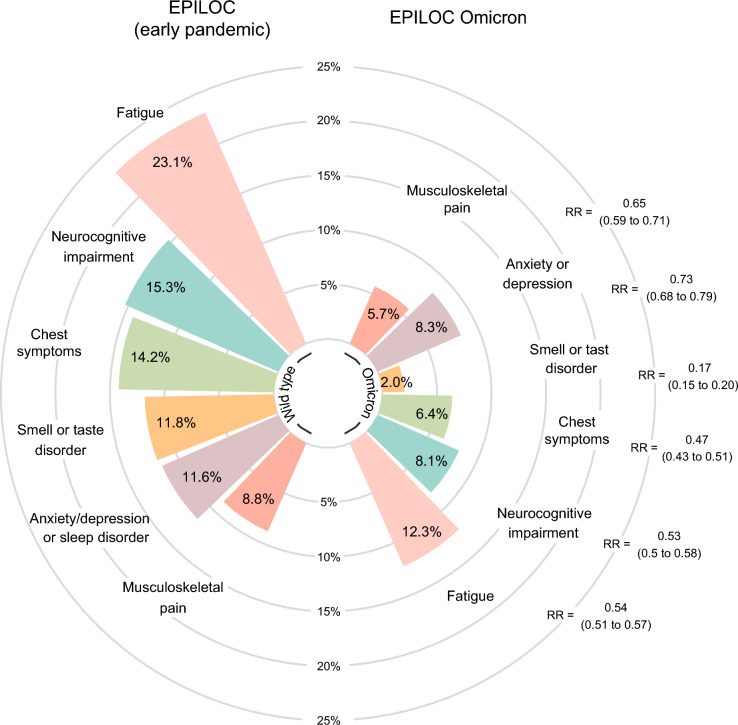
Prevalence of main post-COVID-19 symptom clusters (of grade moderate to strong) after infection during Omicron vs. early pandemic , with age, sex, and education adjusted relative risks with 95%-CI.

As some individual symptoms are strongly correlated, we investigated previously defined post-COVID symptom clusters (Fig. 3). Moderate to strong symptoms of the fatigue cluster were prevalent in 23.1% of participants of EPILOC compared to 12.3% for EPILOC Omicron participants (adjusted relative risk [RR]: 0.54). Similar relative risks for EPILOC Omicron vs EPILOC were observed for the other common symptom clusters (RR = 0.53 for neurocognitive impairment, 0.47 for chest symptoms). The lowest prevalence ratio was found for smell or taste disorder with 2.0% vs 11.8% (RR = 0.17). Information for less common symptom clusters can be found in the supplement (Supplemental Fig. 3). Post-exertional malaise (PEM) was not addressed in EPILOC; however, in EPILOC Omicron, 14.8% of participants reported PEM beyond 14 h after physical or mental exertion.

**Fig. 4:**
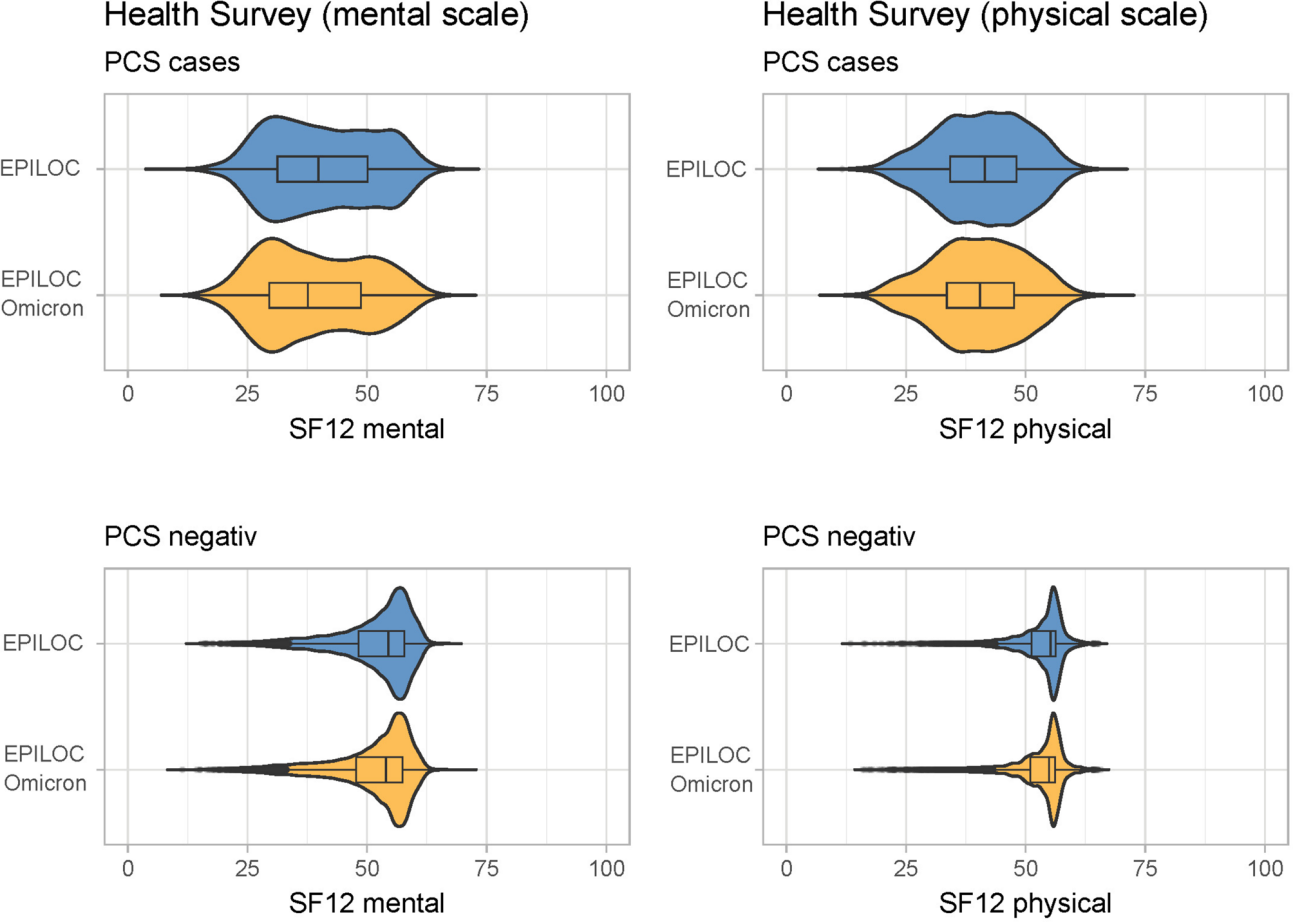
Mental (left) and physical (right) health-related quality of life in participants fulfilling the EPILOC PCS case definition (PCS cases) and those who do not (PCS negative).

Figure 4 shows the distribution of hrQoL scores for both cohorts by EPILOC PCS case status. Mental as well as physical hrQoL were substantially lower in cases compared to PCS-negative participants. However, the distribution of mental and physical hrQoL scores were about the same in PCS cases of both cohorts (mean SF-12_Physical_ 40.3 vs. 41.0, mean SF-12_Mental_ 38.9 vs. 40.7). Adjusted mean differences between PCS cases and non-cases were -11.8 (EPILOC) and -11.3 (EPILOC Omicron) for the physical scale, and -12.3 (EPILOC) and -11.8 (EPILOC Omicron) for the mental scale.

## Discussion

In this large population-based study of SARS-CoV-2-infected adults with mainly mild course of index infection, we found different patterns of acute symptoms after infection during the Omicron era compared to the early pandemic, with some symptoms being more prevalent with Omicron infection (e.g., sore throat, cough, fewer). However, post-COVID-19 symptom prevalences were consistently lower after infection during the Omicron, especially for altered smell or taste, shortness of breath, rapid physical exhaustion, and concentration difficulties. Overall, we found about half the risk for moderate to strong post-COVID-19 symptom clusters of fatigue (RR = 0.54), neurocognitive impairment (RR = 0.53), and chest symptoms (RR = 0.47) several months after Omicron compared to wild-type infection. However, individuals affected by post-COVID-19 symptoms and fulfilling the EPILOC PCS case definition showed equal impairment in hrQoL regardless of the pandemic period.

Changing patterns of acute symptoms with SARS-CoV-2 infection have previously been described. The remarkable decline in loss of smell and taste symptoms was noticed after the Omicron variant emerged^12–14^. Also, increases in the prevalence of sore throat and cough have been observed, while the prevalence of acute phase shortness of breath and fatigue decreased compared to early variants^12^. Some shifts have been observed within Omicron sub-lineages as well; for example, fever was a more likely symptom with BA.5 compared to BA.2 infection^15^.

We found a reduction in PCS risk from 29.6% with wild-type infection in an unvaccinated population to 14.5% with Omicron infection in a mostly vaccinated population (RR = 0.50) when applying the EPILOC PCS case definition. This aligns well with other population-based studies. In an analysis of two population-based Swiss cohorts, Ballouz et al. found that a post-COVID condition is less than half as likely after Omicron infection in a vaccinated population compared to wild-type infection in an unvaccinated population (25.3% vs. 11.1%, OR = 0.42)^16^. Xie et al., using health records of the US Department of Veterans Affairs, found a relative reduction in PASC risk for the Omicron compared to the pre-Delta era of 53–70% of which could be attributed to vaccination and 30% to era-related effects or virus variant^3^. In a large study of vaccine effectiveness against PCS from Sweden they found a risk reduction for adults infected with the Omicron variant through vaccination of 41% (Hazard ratio: 0.59)^17^. Carazo et al. found a relative risk reduction for long-COVID by hybrid immunity due to booster vaccination and prior infection of 65 to 79% using Canadian survey data combined with an immunization registry and administrative data^18^. However, Diexer et al., using data from the German DigiHero platform for health research, found a much larger difference in PCS risk between unvaccinated wild-type compared to vaccinated Omicron, with an OR of 6.4^19^. Hedberg et al. found 6.3 times higher risk for a PCC diagnosis (ICD-10 code U09.9) after wild-type compared to Omicron infection bases on linkage of population based data sources^20^. Differences might be related to population characteristics or the timing of the assessment; Diexer et al. defined PCC as at least one symptom 12 weeks after infection, while Ballouz et al. evaluated symptoms six months after assessment, and Xie et al. assessed incident health outcomes between 30 days and one year after infection. The risk reduction might also be affected by the PCS case definition used. Hedberg et al. only included cases that sought medical care and obtained a PCC diagnosis. We found a less pronounced risk reduction for PCS after infection during the Omicron era than during the early pandemic, when applying the COVIDOM case definition (RR = 0.66). The COVIDOM case definition places less weight on smell and taste disorders, which were less dominant after Omicron infection, and more weight on upper respiratory symptoms, which were more dominant after Omicron infection. When using at least one new moderate-to-strong-intensity symptom to define a PCS case, we found a 40% risk reduction after infection during the Omicron era compared with the early pandemic (RR = 0.60).

Predictors of PCS were, however, very consistent and independent of the PCS case definition, and they were similar for PCS after in both phases of the pandemic. Treatment of acute infection, as proxy of more severe acute infection, was strongly associated with PCS after infection during both pandemic periods. Likewise, women, obese participants, smokers and participants with a history of chronic diseases were at higher PCS risk. All these risk factors have also been previously described for different SARS-CoV-2 variants^21–24^.

Independent of the pandemic period, we found hrQoL (physical and mental) to be impaired in those affected by PCS, with an about 11 to 12 points lower SF12 score compared to individuals not fulfilling the EPILOC PCS case definition. For comparison, a difference in ≥ 4 points in the physical or mental scale is considered clinically relevant^25^. In fact, the on average low physical hrQoL scores in PCS cases are comparable to patients with chronic obstructive pulmonary disease GOLD grade III^26^. and the average low mental hrQoL scores are in the range of patients with anxiety disorders^27^. Our results regarding hrQoL are in line with other studies; e.g., Beyer et al. reported mean SF-12 scores of 35.2 for the physical and 40.9 for the mental scale in 69 patients with diagnosed PCS at a university hospital^28^. Wallace et al. studied 59 individuals with pre-existing rheumatic disorders and long-COVID and found a mean physical score of 37.7 and a mean mental score of 45.3. Hopff et al. found comparable hrQoL in patients three months after Delta or Omicron infection^29^. Jacka et al. found substantial hrQoL impairment in Long-Covid affected individuals for up to 24 months post infection in the pre-Omicron era^30^. However, population-based data on hrQoL in PCS is scarce.

Strengths of our study include the population-based design and the large sample size. We applied multiple PCS definitions, including an externally developed definition (COVIDOM) and a symptom-based definition, and observed consistent patterns across all approaches. We used a similar study design, the same target population in the same study region, and similar questionnaires for both cohorts, thereby enhancing comparability. However, some differences exist; during the Omicron wave, infections were less likely to be confirmed by PCR or antigen due to limited resources. Response rates were lower for EPILOC Omicron compared to EPILOC (17% vs 24%). As individuals who have completely recovered might be less likely to participate, this could lead to an underestimation of the risk ratio for PCS after Omicron infection compared to wild-type infection. The average time since infection was longer for the EPILOC Omicron assessment compared to EPILOC (15.1 months vs. 8.5 months). However, a follow-up assessment of the EPILOC cohort, on average 24.2 months after infection, found little change in the net PCS prevalence compared to 8.5 months after infection (see also Supplemental Fig. 4)^31^.

## Conclusions

PCS was less common following SARS-CoV-2 infection in the Omicron era compared to the early pandemic period. However, affected individuals continue to experience substantial, comparable impairment in hrQoL across both pandemic periods.

## Supplementary Information

Supplementary Information.

## Data Availability

The EPILOC consortium has established a Data Access and Use Committee; requests may be sent to dauc.epiloc@uni-ulm.de.
