# Supplementary material for: Population-based comparison of post-acute sequelae of COVID-19 and health-related quality of life across pandemic periods: Omicron era versus early pandemic
Source: Sci Rep. 2026 May 19;16:15603. doi: 10.1038/s41598-026-52945-2 (PMC13187147; doi:10.1038/s41598-026-52945-2)
Supplement: Supplementary file 1 — Supplementary Information. [file 41598_2026_52945_MOESM1_ESM.pdf]

Supplementary material

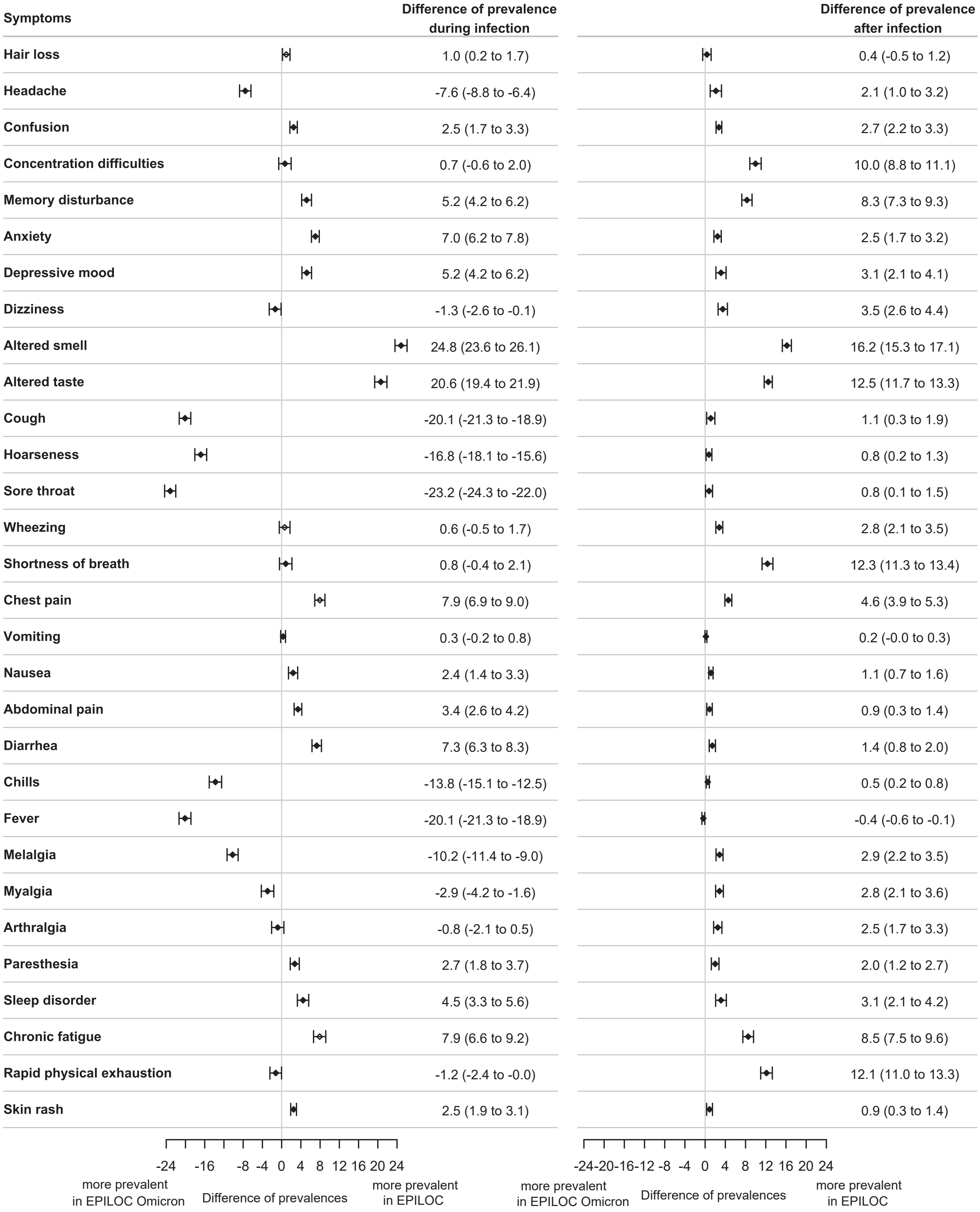

Supplemental Figure 1. Age, sex, and education adjusted differences (with 95% confidence interval) of symptom prevalence (EPILOC Omicron vs. EPILOC) during acute COVID-19 (left panel) and post-COVID-19 (right panel).

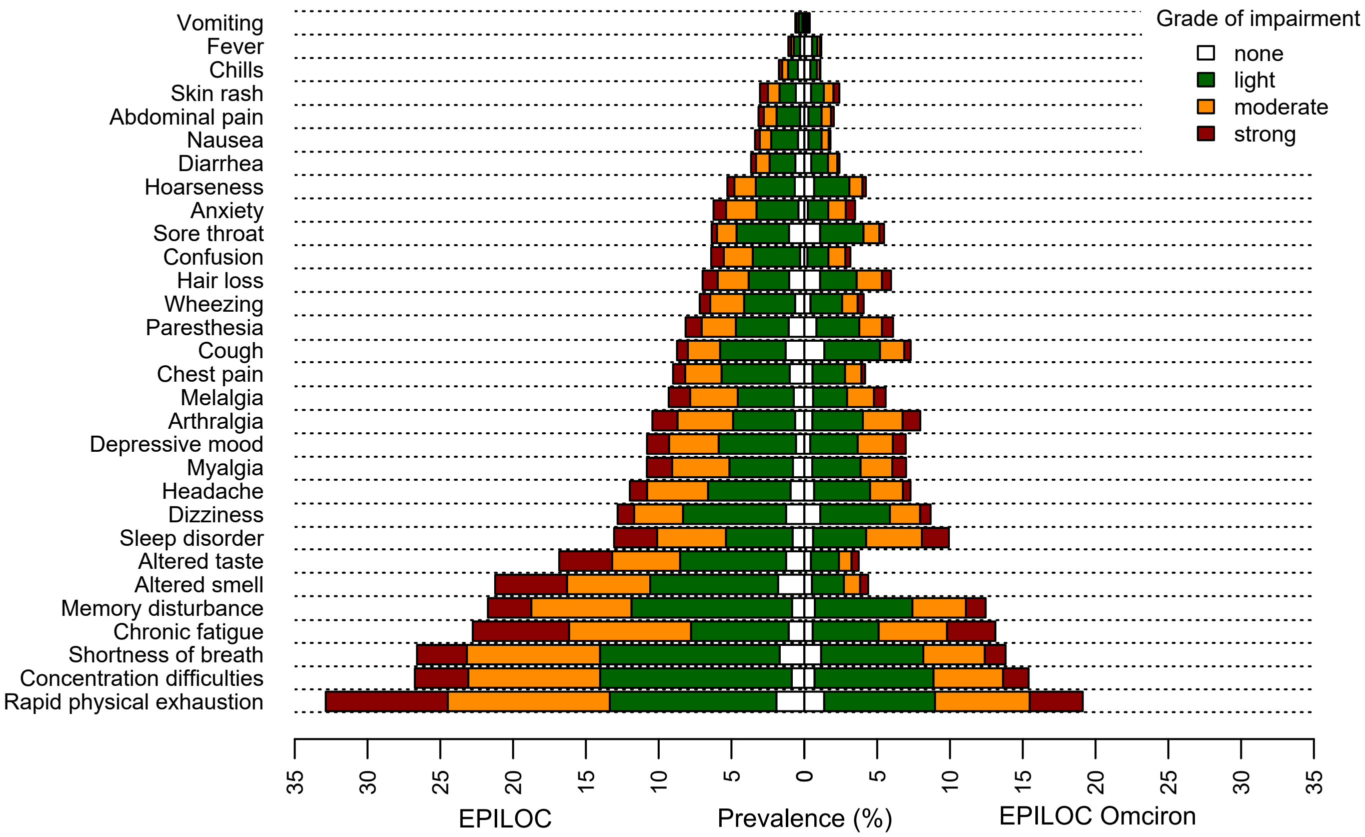

Supplemental Figure 2. Prevalence of post-Covid Symptoms in EPILOC and EPILOC Omicron with grade of impairment.

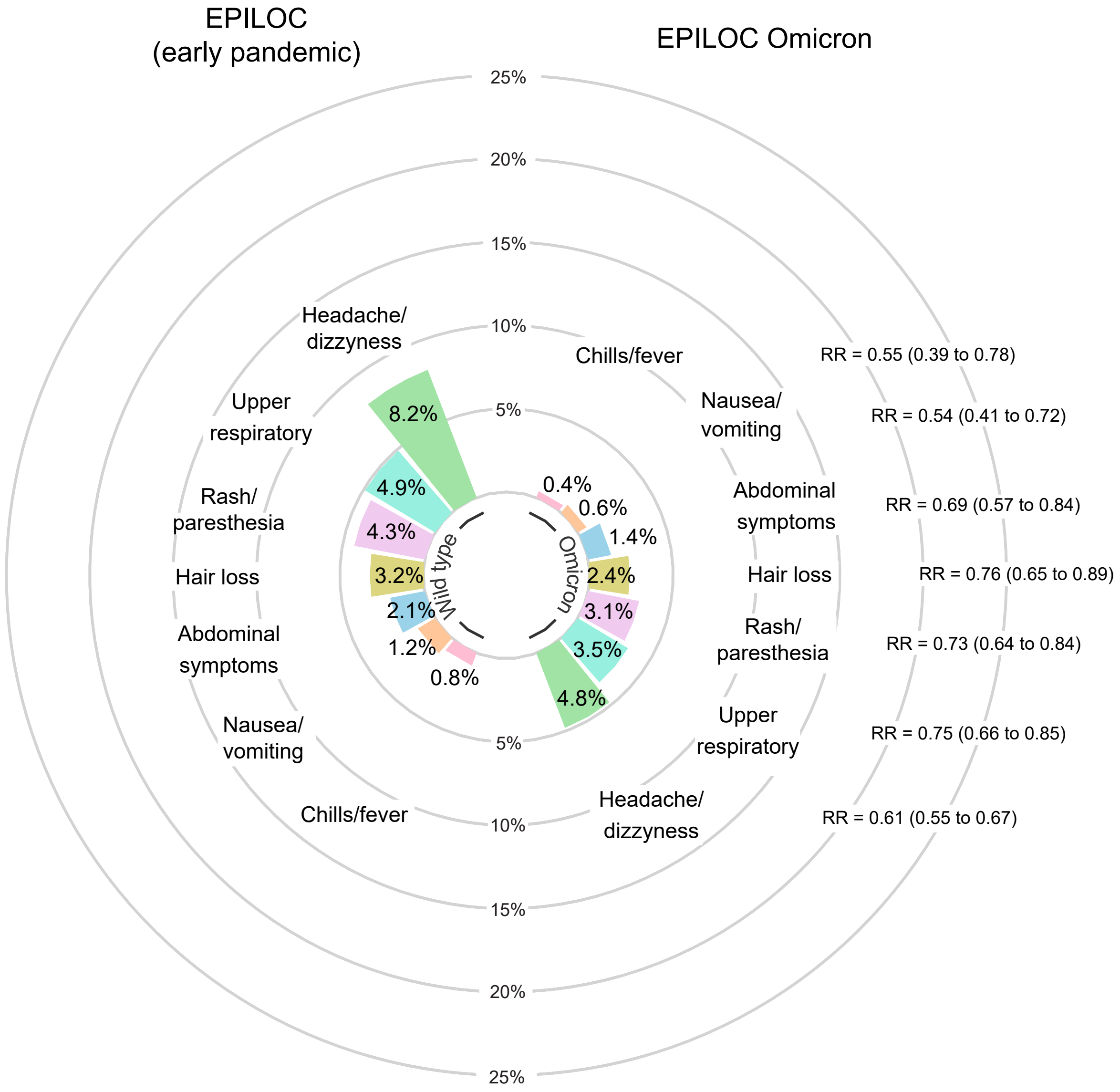

Supplemental Figure 3. Prevalence of less common post-COVID-19 symptom clusters (of grade moderate to strong) in EPILOC Omicron vs. EPILOC, with age, sex, and education adjusted relative with 95%-CI.

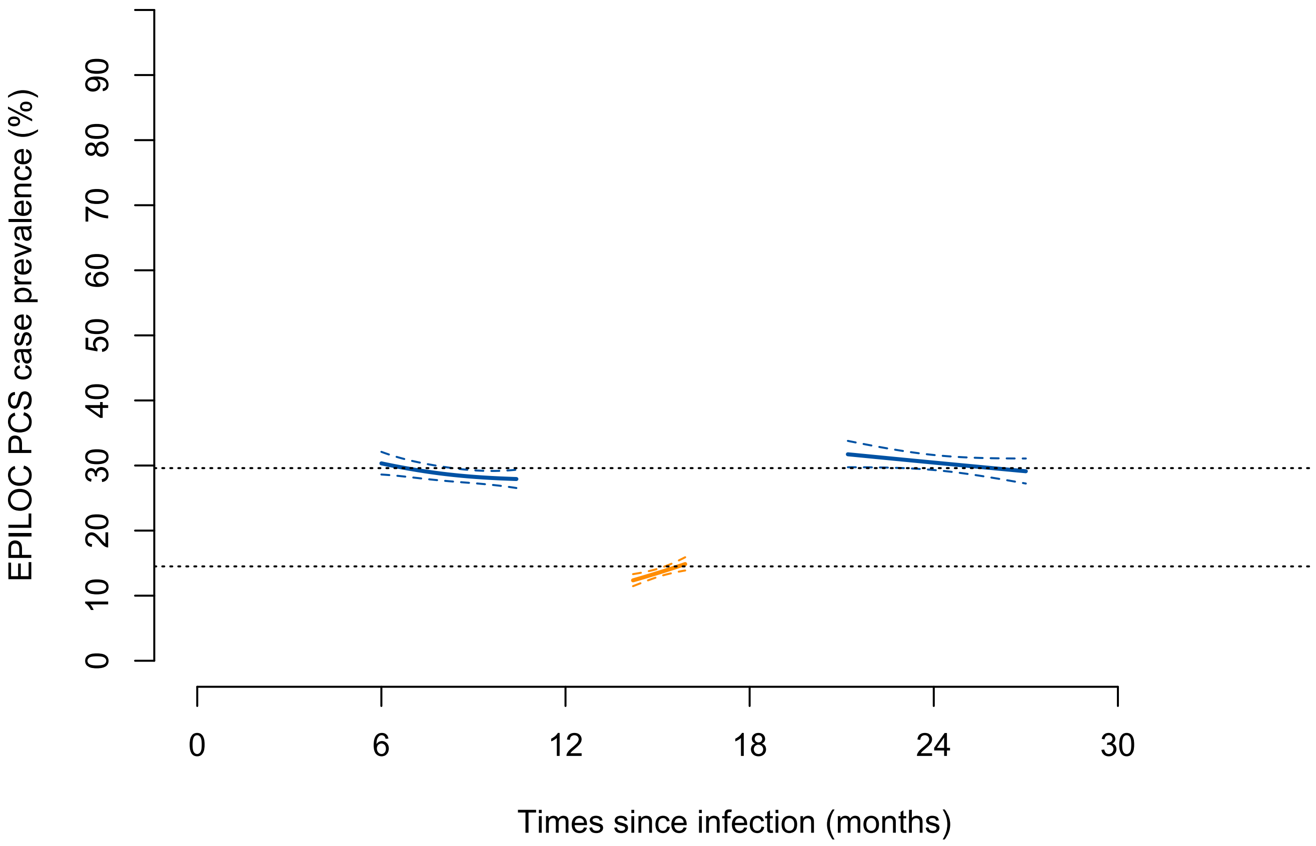

Supplemental Figure 4. PCS prevalence according to the EPILOC case definition over time since infection in the EPILOC (early pandemic index infection) assessments (blue) and EPILOC-Omicron (orange), adjusted for sex, age, education and smoking.
